# Betalain–Chickpea Protein Particles Produced by Freeze Drying and Spray Drying: Physicochemical Aspects, Storage Stability, and In Vitro Digestion

**DOI:** 10.3390/foods14020281

**Published:** 2025-01-16

**Authors:** Mary H. Grace, Roberta Targino Hoskin, Malak Alghamdi, Mary Ann Lila, Vesela I. Chalova

**Affiliations:** 1 Plants for Human Health Institute, Department of Food, Bioprocessing and Nutrition Sciences, North Carolina State University, 600 Laureate Way, Kannapolis, NC 28081, USA; mhgrace@ncsu.edu (M.H.G.); rtcorrei@ncsu.edu (R.T.H.); malgham@ncsu.edu (M.A.); mlila@ncsu.edu (M.A.L.); 2 Department of Biochemistry and Nutrition, University of Food Technologies, 4002 Plovdiv, Bulgaria

**Keywords:** plant based, pigments, protein-rich particles, valorization, delivery method

## Abstract

Beetroots are one of the primary sources of betalains, nitrogenous pigments with anti-inflammatory and antioxidant properties. However, due to their chemical instability, betalains have limited use in food applications. This work investigated whether betalains encapsulated in chickpea protein could be stabilized and delivered in a shelf-stable format. Freeze-dried (CB-FD) and spray-dried (CB-SD) protein–betalain particles encapsulated in chickpea protein isolate (6% *w*/*v*) were prepared. The encapsulation method affected particles’ morphology, water activity, hygroscopicity, solubility, and color. Particles captured total betalains of 9.30 ± 0.61 and 4.40 ± 0.92 mg/g for CB-SD and CB-FD, respectively. LC-MS identified 12 betacyanins and 6 betaxanthins. The stability of betalains revealed that encapsulation efficiently preserved betalain integrity of over 6 weeks of storage at 4, 22, and 40 °C compared to dry beetroot extract. CB-SD particles were stable with no significant changes, while CB-FD showed slight degradation after 4 weeks due to increased A*_w_*. Antioxidant activity correlated well with betalain concentration. In vitro digestion resulted in only 25% bioaccessibility of betacyanins, while betaxanthins were more stable with 100% recovery. Encapsulation with chickpea protein isolate is an efficient and straightforward strategy for expanding and diversifying applications of phytochemical-rich beetroot extracts for the food industry.

## 1. Introduction

Beetroots (*Beta vulgaris*, family *Chenopodiaceae*) are vegetables produced worldwide throughout the seasons and are one of the main natural sources of betalains, as well as phenolic compounds, vitamins, carotenoids, and minerals [1]. Betalains are water-soluble nitrogenous pigments composed of reddish-violet betacyanins and yellowish-orange betaxanthin pigments with documented biological effects, particularly in the prevention and management of chronic conditions such as diabetes, obesity, and cardiovascular diseases [1].

However, the use of beetroots in food formulations is challenged by their earthy smell and taste and natural perishability [2]. Moreover, betalains are heat-sensitive molecules and susceptible to degradation caused by pH fluctuations, oxygen and light, and the presence of metal cations [3]. Therefore, creative solutions to enable longer shelf life, expand the portfolio of products, and provide value-added methods for the use of beetroots in the food industry are needed [4].

Encapsulation techniques have been intensively studied as technologically sound methods for improving stability, preserving biological activity, and ensuring easier handling and transportation of food products. Spray drying is one of the most popular encapsulation techniques in the food industry today [5,6,7], while freeze drying, although generally more expensive than spray drying, is known for yielding well preserved, high-quality compounds [8].

Our group explored encapsulation techniques to obtain protein–polyphenol particles with preserved phytochemicals, enhanced storability, and desirable technological attributes [9,10], for multiple food applications. In this work, we used our well-established processing strategy to investigate and compare spray drying and freeze drying techniques as technological routes to produce protein–betalain ingredients using chickpea protein isolate and betalain-rich extracts from beetroots. The resulting particles produced by spray drying and freeze drying were comprehensively analyzed in terms of physicochemical attributes, betalain content, profile, storage stability, and in vitro bioaccessibility. The outcomes of this study provide practical information on the processing of abundant yet underexplored phytochemicals from beetroots to deliver safe, stable, and marketable food products.

## 2. Materials and Methods

### 2.1. Materials

Fresh organic red beetroots (Merlin F1-hybrid, *Beta vulgaris* L. subsp. vulgaris var. conditiva Alef.) provided by a local grower were stored in a cold room (4–6 °C) and processed within two weeks. Chickpea protein isolate (ChickP S930) was obtained from ChickP Protein Ltd. (Rehovot, Israel) and contained 89.7% protein, 7.0% total carbohydrates, and less than 0.1% fat (according to the manufacturer’s specifications). Solvents used in high-performance liquid chromatography (HPLC) or liquid chromatography-mass spectrometry (LC-MS) studies were of ACS or HPLC grade. All remaining reagents were of analytical grade. Distilled or double-distilled water was used to prepare the solutions.

### 2.2. Preparation of Beetroot Extract

Beetroots were washed and cut into small pieces that were mixed with 80% aqueous ethanol solution in a laboratory blender 100:400 *w*/*v* (Vitamix, Vita-Mix Corporation, Cleveland, OH, USA), then processed under vacuum for 5 min at room temperature, followed by vacuum filtration through cheesecloth and centrifugation (Sorvall Legend RT, Thermo Electron Corporation, Osterode, Germany) at 4000 rpm, 10 °C for 20 min. The supernatant was collected and vacuum concentrated in a rotary evaporator at 46 °C (Rotavapor R-210, BUCHI Corporation, New Castle, DE, USA) until complete evaporation of ethanol and concentration to yield an aqueous concentrated beetroot extract (°Brix 12.5 ± 0.5, pH 5.8 ± 0.5). The concentrated extract was collected, stored in dark containers at 4 °C, and used to prepare chickpea protein–betalain particles within two days.

### 2.3. Freeze Drying (FD) and Spray Drying (SD) Processes

Before drying, the concentrated beetroot extract was mixed with water in a 2.5-fold dilution, resulting in a solution with a total betalain concentration of 9.075 mg/g dw. To identify the optimal combination of chickpea protein and beetroot extract that maximized betalain content, preliminary experiments were conducted (Tables S1 and S2). A 6% *w*/*v* chickpea solution, determined during these initial tests, was combined with beetroot solution and stirred for 30 min at room temperature until complete homogenization was achieved. For FD, the beetroot extract-chickpea protein suspension was centrifuged for 20 min at 4000 rpm. The supernatant containing the excess beetroot extract was discarded, and the pelleted material was collected, and subjected to −20 °C for 12 h before being FD. The FD procedure lasted 48 h (Labconco, Labconco Corporation, Kansas City, MO, USA). The condenser temperature was −55 °C, and the vacuum pressure was 0.105 Torr. The dried material was pulverized using an IKA A11 analytical mill (Wilmington, NC, USA), placed in tightly closed containers, and stored in a −20 °C freezer for further analysis.

The SD was carried out based on our protocol [11] using a lab-scale spray dryer (model B-290, Buchi Labortechnik AG, Flawil, Switzerland) using a 0.7 mm nozzle, 7.5 mL/min of feed flow controlled by a peristaltic pump, aspirator rate at 100%, with air in co-current flow. The feed solutions prepared by mixing chickpea protein and beetroot extracts were kept under constant stirring at room temperature during the spray drying process. The drying inlet air temperature was set to 125 °C, and the outlet air temperature was 64–66 °C. Spray-dried protein–betalain particles were collected from the powder collection vessel only, weighed, and stored in sealed plastic containers at −20 °C until further use. The SD yield was calculated according to Hoskin et al. [10]. Freeze-dried and spray-dried chickpea protein–betalain particles were labeled as CB-FD and CB-SD, respectively.

### 2.4. Characterization of Chickpea Protein–Betalain Particles

#### 2.4.1. Water Activity (A*_w_*)

The water activity (A*_w_*) was determined by using a water activity meter (Aqualab 4TE, Meter Group, Inc., Pulman, WA, USA).

#### 2.4.2. Hygroscopicity and Solubility

Hygroscopicity and solubility analyses were determined according to Correia et al. [12]. For hygroscopicity, samples (0.5 g) were placed in a desiccator containing a saturated solution of NaCl (RH 75.3%). Results were expressed as the mass of water absorbed per 100 g of sample after 7 days of storage. For solubility, samples (0.5 g) were mixed with 50 mL of distilled water and vigorously homogenized at high velocity for 5 min and centrifuged at 4000 rpm for 5 min. An aliquot (25 mL) of the supernatant was removed, transferred to aluminum dishes, and dried to constant weight in an oven at 105 °C. Results expressed as a percentage (%) were calculated as the ratio between the weight of the supernatant (soluble solids in solution) and the weight of the sample.

#### 2.4.3. Instrumental Color Evaluation

Color measurements were performed using a CR-5 colorimeter (Konica Minolta, Tokyo, Japan). The CIELAB L* (lightness), a* (redness-greenness), and b* (yellowness-blueness) parameters were recorded and used to calculate total color difference (ΔE) [13]. The colorimetric analyses were performed in triplicate.

#### 2.4.4. Morphology by Scanning Electron Microscopy (SEM)

The morphological analysis was conducted using a scanning electron microscope (SU3900, Hitachi, Tokyo, Japan). Samples were placed on a conductive carbon tape and analyzed under a vacuum. The morphological analysis was conducted at an accelerating voltage of 20 kV and 250, 500, and 1000× magnifications.

### 2.5. Phytochemical Analyses

#### 2.5.1. Elution of Phytochemicals

Protein–betalain particles were sampled (0.1 g) and mixed with 4 mL 80% aqueous methanol solution. The extraction of phytochemicals was performed by sonication for 5 min at 30 °C followed by centrifugation (12,000 rpm, 10 min). The procedure was repeated 2 times. The supernatants were pooled together and transferred to a 10 mL volumetric flask. The volume was leveled with extraction solvent. The eluents were used for betalain and antioxidant capacity assessments.

#### 2.5.2. Betalain Content by Spectrophotometry

Spectrophotometric evaluation of betalain content was performed as described by Šaponjac et al. [14]. Briefly, absorbance readings of liquid samples were taken by a microplate reader (SpectaMax Plus 384, Molecular Devices, LLC., San Jose, CA, USA) at 538 (a) and 476 nm (b) for evaluation of betacyanin and betaxanthin, respectively. A third measurement at 600 nm (c) was taken to correct for colored impurities. The absorbance of betanin (x) and vulgaxanthin-I (y) corrected for colored impurities were calculated using the following equations:

x = 1.095 × (a − c)

y = b − z − x/3.1

z = a − x

where z is the absorbance of impurities. The concentrations of betacyanin (in terms of betanin) and betaxanthin (in terms of vulgaxanthin-I) were calculated by using the following equation:

C (mg/100 mL) = x(y) × F × 1000/A^1%^,

where F is the dilution factor, and A^1%^ is the absorbance coefficient for betanin (1120) or violaxanthin-I (750). The contents of betacyanin or betaxanthin in the protein–betalain particles were expressed as betanin or vulgaxanthin-I equivalents (mg BE or VE/g sample).

#### 2.5.3. HPLC Profile for Betalains

The analysis of betacyanins was conducted using an Agilent 1200 HPLC (Agilent Technologies, Santa Clara, CA, USA) equipped with a photodiode array detector (DAD). Separation of the compounds was carried out on Phenomenex Synergi 4 μm hydro-RP 80A column (250 mm × 4.6 mm × 5 μm, Torrance, CA, USA) with a constant flow rate of 1 mL/min at 30 °C. The elution was conducted using a binary solvent system consisting of solvent A (5% aqueous formic acid) and solvent B (100% acetonitrile) and a gradient as follows: 0% B (0–20 min), 30% B (20–25 min), 70% B (25–27 min), 80% B (27–29 min), 0% B (29–35 min). Spectra were collected at 532 nm for betacyanins and at 480 nm for betaxanthins. Betacyanins in the samples were identified by matching the retention time and spectral characteristics of betanin and isobetanin in red beet extract diluted with dextrin (dissolved in 50% methanol).

### 2.6. Antioxidant Activity—DPPH Assay

The antioxidant activity was evaluated using the 2,2-diphenyl-1-picrylhydrazyl radical scavenging method (DPPH assay) [15]. Briefly, a 20 µL sample was mixed with 180 µL DPPH solution (150 µM in methanol 80%) in a microplate. Incubation for 40 min in the dark (room temperature) was followed by an absorbance measurement at 515 nm. Trolox (6-hydroxy-2,5,7,8-tetramethylchroman-2-carboxylic acid) was used to generate a standard curve. The results were expressed in Trolox equivalents (µmol TE).

### 2.7. Storage Stability

Protein–betalain particles and FD BEx were evaluated during a 6-week storage period. For this, samples were placed in opaque vials and stored in the dark at 5, 22, and 40 °C. Samples were analyzed right after production (week 0) and after 2 (week 2), 4 weeks (week 4), and 6 weeks (week 6) of storage for the determination of betalain content and antioxidant activity (measured as DPPH radical scavenging activity). Water activity and color evaluation, including L*, a*, b*, and total color difference (ΔE), were analyzed at week 4 and calculated according to Zhang et al. [16].

### 2.8. In Vitro Gastrointestinal Digestion Bioaccessibility Assay

This study’s in vitro gastrointestinal digestion (GID) method was adapted from [17]. Initially, simulated salivary fluid (SSF), simulated gastric fluid (SGF), and simulated intestinal fluid (SIF) electrolyte stock solutions were prepared with the corresponding electrolytes. CB-FD and CB-SD particles (n = 3 replicates) were normalized to a concentration of 10 mg/g total betalains. For the oral phase, samples were suspended in 1 mL of distilled water, mixed with 0.7 mL of SSF, followed by sequential addition of 100 µL of 1500 U/mL porcine pancreas α-amylase solution, 50 µL of 0.03 M CaCl_2_, and 150 µL of water (pH 7.0), homogenized for 2 min. The gastric phase started with mixing the 2 mL oral bolus with 0.64 mL of SGF, 160 μL porcine pepsin solution (25,000 U/mL), 5 μL of 0.03 M CaCl_2_, and the pH was adjusted to 3.0 with HCl. Distilled water was added to achieve a total volume of 4 mL followed by agitation at 37 °C for 2 h (Innova 44r Incubator Shaker Series, New Brunswick Scientific, Edison, NJ, USA). For the intestinal stage, 4 mL of gastric chyme was mixed with 2.2 mL of SIF, 1 mL of a pancreatin solution (800 U/mL), 500 μL fresh bile (160 mM in fresh bile), 80 μL of 0.03 M CaCl_2_, and the solution was adjusted to pH 7.0 with NaOH. Distilled water was added to achieve a total volume of 8 mL, and the mixture was shaken for an additional 2 h at 37 °C. Samples were centrifuged to obtain the soluble fraction (supernatant) and the residual fraction, which were immediately frozen and freeze-dried. The bioaccessibility index (BI) for betacyanidins and betaxanthins was calculated according to

$$BI\%=\left(\frac{A}{B}\right)*100$$

where *A* corresponds to the metabolite (betalain) contents (mg/g) quantified in the intestinal supernatant residues for CB-FD or CB-SD, and *B* is for the initial mg/g betalain content for CB-FD, or CB-SD, quantified pre in vitro digestion.

### 2.9. Statistical Analysis

Data are presented as mean values and standard deviation of experiments performed in triplicates unless specified otherwise. Prism 10.0.3 (GraphPad Software, Boston, MA, USA) was used to perform the analysis of variance (ANOVA) with *p* < 0.05 using Tukey’s or Dunnett’s multiple comparisons tests. An unpaired *t*-test was used for comparison between two groups.

## 3. Results and Discussion

### 3.1. Characterization of Protein–Betalain Particles

#### 3.1.1. Visual Aspect and Morphology

**Figure 1:**
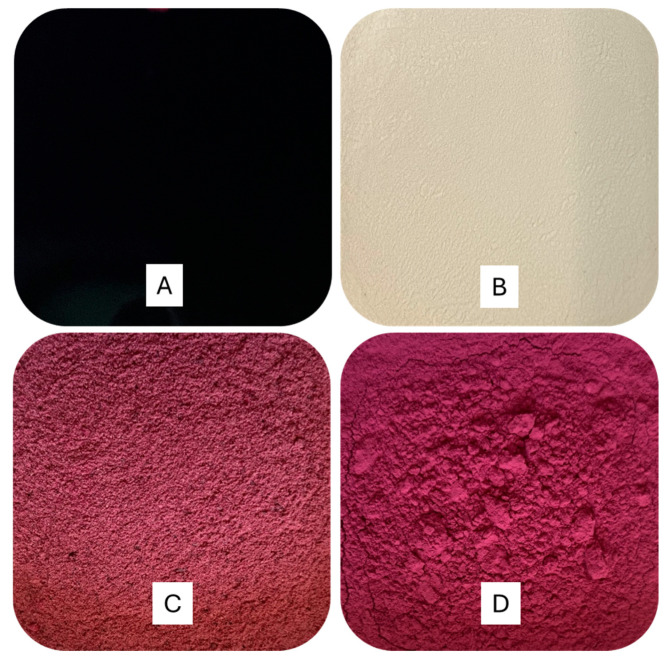
Concentrated beetroot extract (**A**), chickpea protein isolate (**B**), freeze-dried chickpea protein–betalain particles (**C**) and spray-dried chickpea protein–betalain particles (**D**).

The beetroot extract had a bright red color, while chickpea protein is a whitish fine powder. CB-SD particles showed a bright reddish color and the aspect of a fine powder, which is typical for spray-dried powders. In contrast, CB-FD particles had a lighter pinkish-red color than CB-SD, and particles had a rough, granular aspect (Figure 1).

**Figure 2:**
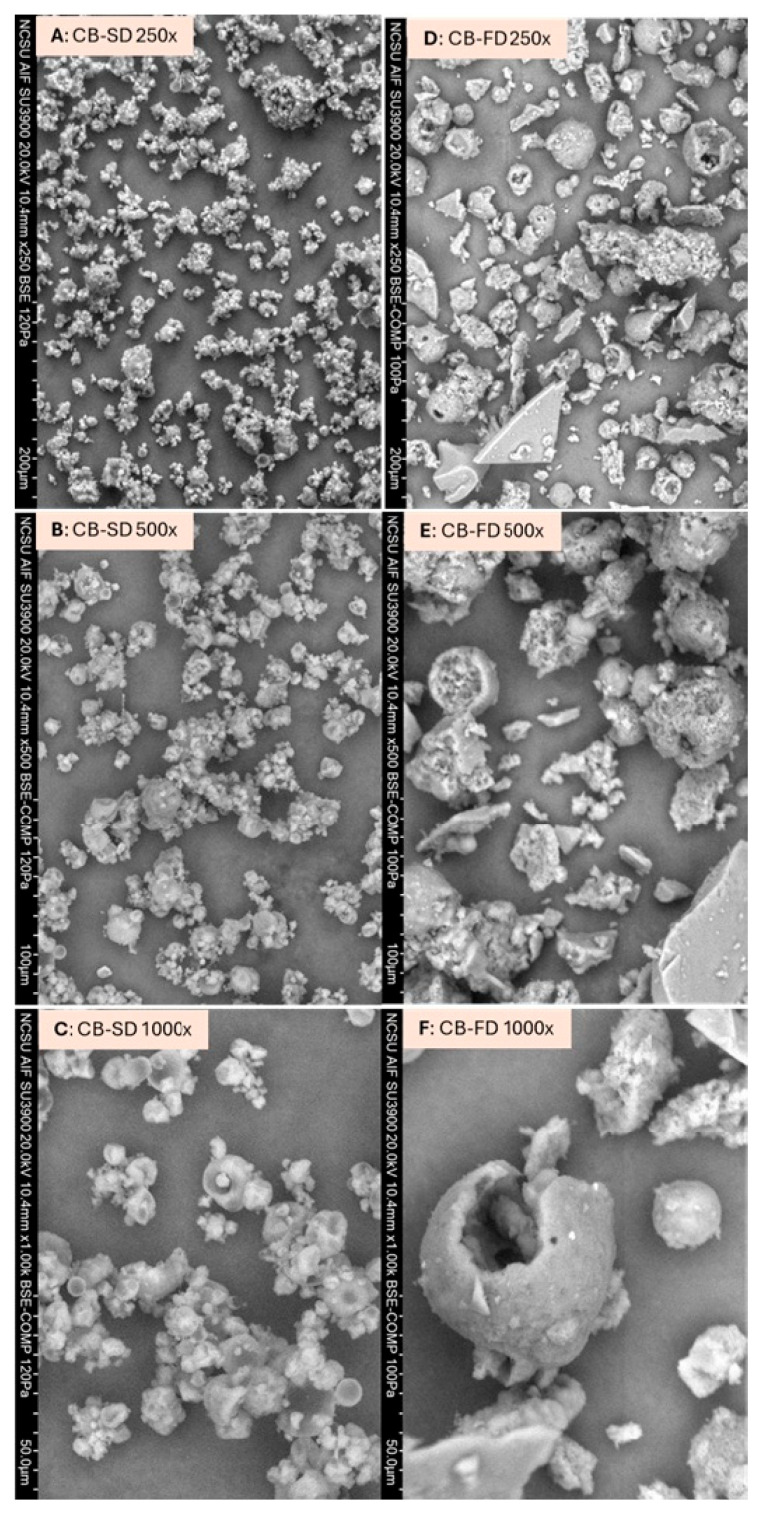
Scanning electron micrographs of CB-SD: spray-dried chickpea protein–betalain particles (**A**–**C**) and CB-SD: freeze-dried chickpea protein–betalain particles (**D**–**F**). Images (**A**,**D**): magnification 250×; images (**B**,**E**): magnification 500×; images (**C**,**E**): magnification 1000×.

SEM results show the impact of each encapsulation technique on particle structure. While CB-SD produced spherical particles of various sizes with some shrinkage and concavities, CB-FD particles were mostly characterized by irregular shapes with visible pores and edges (Figure 2). In addition, CB-FD particles showed plate-shaped, fractured structures resembling the aspect of broken glass, while CB-SD particles had smoother surfaces. This is explained by the forced passage through the atomization nozzle of the spray dryer, which induces the formation of more regular spherical shapes for spray-dried particles [12]. On the other hand, FD induces the formation of porous structures due to ice crystal formation during freezing and the sublimation process during drying [18]. When comparing images with the same magnification, CB-SD particles are visibly smaller than CB-FD. Similar morphological observations were made when comparing spray-dried and freeze-dried particles derived from blackberry juice [19], beetroot juice [20], and moringa extracts [13]. These morphological differences may affect the stability of food particles. For example, particles with significantly fractured surfaces might lead to higher oxygen permeability during storage and consequent higher core material degradation [21].

#### 3.1.2. A*_w_*, Solubility, and Hygroscopicity

CB-SD samples had an A*_w_* value (0.2868) typically reported in the literature for spray-dried samples. In contrast, CB-FD had a significantly lower A*_w_* (0.0436; *p* < 0.05; Table 1), which agrees with the observed granular, crispy texture of freeze-dried samples. Water activity depends on the applied operational conditions, and it is strongly correlated with food texture. A similar trend was observed for spray-dried and freeze-dried beetroot juice with pumpkin protein [22]. Despite the observed differences, both treatments are within the microbiologically safe range (A*_w_* < 0.65) for food products [21].

A fast and complete powder reconstitution is a critical quality aspect. Indeed, when it comes to food formulation and incorporation of food powders into food products, higher solubility is a desirable attribute for most industrial operations since it simplifies production protocols and minimizes preparation time and costs [14]. Significant differences were observed for solubility in water: while CB-SD was highly soluble (almost 80%), CB-FD had poor solubility (approx. 52%; Table 1). The tested solubility of chickpea protein in water was 39.75%. Spray-dried samples are smaller and more uniform particles (Figure 2) than freeze-dried particles, which might play an important role in the observed differences [23]. It was found that chemical interactions between protein and polyphenols may result in protein–polyphenol structures with either higher or lower solubility, depending on the type of protein, polyphenol, and pH [24]. In our study, both CB-SD and CB-FD particles had higher solubility than chickpea protein alone (39.95 ± 0.75%) when evaluated in their natural pH (approx. pH 6.5). Higher protein surface hydrophilicity induced by phenolic binding might play a role in the observed higher solubility of CB-SD and CB-FD compared to non-complexed chickpea protein [25].

In addition, the drying method significantly affected the hygroscopicity of resulting protein–betalain particles (*p* < 0.05; Table 1). The observed difference results lead to distinct definitions according to GEA classification [23], while CB-FD is classified as slightly hygroscopic (results between 10% and 15%), and CB-SD is considered hygroscopic (results between 15% and 20%). A similar trend was observed for moringa leaf extract with maltodextrin and high methoxyl pectin [13] and beetroot juice with pumpkin protein encapsulated by spray drying and freeze drying [20]. Both treatments had higher hygroscopicity when compared to the chickpea protein alone (5.17 ± 0.06%), which indicates that the increased capacity to absorb water from the environment is directly related to the presence of the beetroot extract solids. Our results are higher than spray-dried and freeze-dried protein–polyphenol particles produced with wild blueberry pomace extracts and different protein (soy protein isolate, chickpea, and wheat flour) sources [12] but lower than spray-dried elderberry juice and pomace extract with soy protein isolate [24].

#### 3.1.3. Phytochemical Composition

Betalains were measured using the photometric method. The recorded concentration of red betacyanins pigments is 12.37 ± 0.85 mg BE/g for the beet extract (BEx), 2.59 ± 0.12 mg BE/g for freeze-dried concentrated beet (CB-FD), and 5.48 ± 0.36 mg BE/g for spray-dried concentrated beet (CB-SD). For the yellow-orange pigments called betaxanthins, the measurements were 7.68 ± 0.71 mg VE/g for BEx, 1.84 ± 0.19 mg VE/g for CB-FD, and 3.84 ± 0.29 mg VE/g for CB-SD. In total, the betalain content was 19.85 ± 1.54 mg/g dry weight (dw) for BEx, 4.40 ± 0.92 mg/g dw for CB-FD, and 9.30 ± 0.61 mg/g dw for CB-SD (Table 1). The DPPH antioxidant capacity assay recorded 82.95 ± 10.00 and 64.75 ± 4.20 µMol Trolox equivalent for CB-SD and CB-FD, respectively.

The betalain content in CB-FD was found to be lower than that in CB-SD, as shown in Table 1. This difference can be attributed to the complexation method employed in preparing the CB-FD complex. Specifically, during this process, centrifugation was used, which effectively separated the supernatant rich in excess betalains from the pellet containing the betalain–protein complex. Subsequently, the pellet was freeze-dried to produce the final CB-FD particles [9]. Moreover, spray drying is a drying technique that uses a high temperature-short time principle. Due to the evaporative cooling effect, the particles are exposed to a much lower processing temperature than the inlet temperature, which means that the exposure to high temperatures is minimal. In fact, drying takes place almost instantaneously and an intensive evaporation happens at the surface of each droplet [26,27]. Other studies have shown similar trends. For example, compared to freeze drying, spray drying showed a better or similar performance regarding the retention of betalains and phenolics from red cactus pear [28].

**Figure 3:**
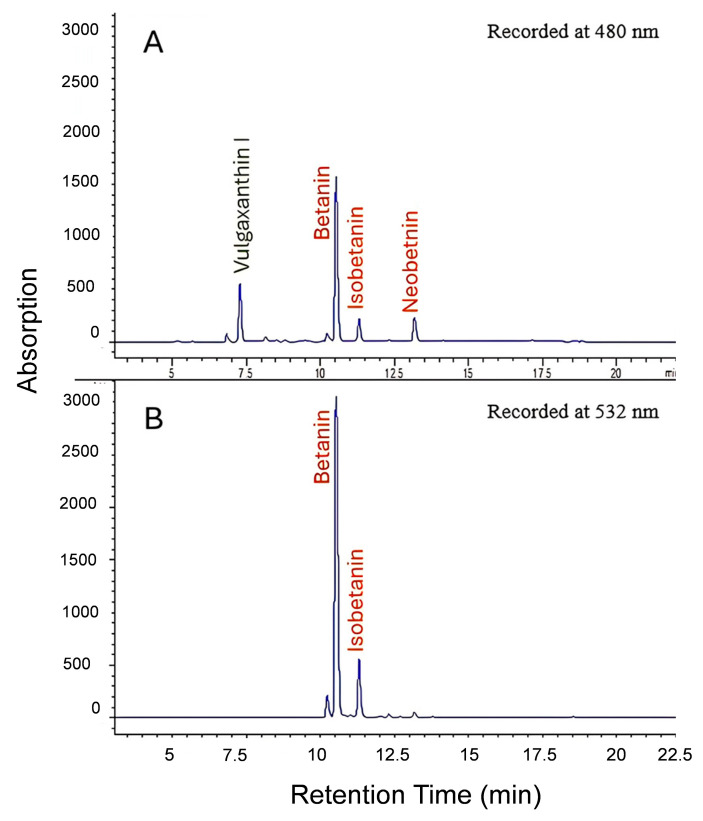
HPLC profiles of betalains recorded at 480 nm showing the major betxanthin (Vulgaxanthin I) and major betacyanins (Betanin, Isobetanin and Neobetanin) (**A**) and betacyanins (Betanin, Isobetanin) recorded at 532 nm (**B**).

HPLC-DAD detection of major betalains recorded at 480 and 532 nm wavelengths compared to reference standard beet root extract displayed profile similarity. Three betacyanins were identified: betanin, isobetanin, and neobetanin. Vulgaxanthin-I showed up at 480 nm (Figure 3). Betanin is the major component in red beetroot. HPLC peak areas showed that the ratio of betanin to violaxanthin-1 is 1.0:0.2.

LC-MS and MS2 allowed us to predict the molecular formula. With the aid of UV/Vis spectra and retention time, the identification process was accomplished by comparing the experimental MS data to previous studies [29,30,31,32]. Twelve betacyanins, including isomers of betanin, betanidin, and 17-decarboxybetanin, and six betaxanthins were identified in CB-FD and CB-SD particles (Table 2).

There is no commercially [33]. However, the profiles of the compounds could change during the purification process and degradation is possible.

Nemzer [29], using their proprietary method of large- available betalain standards for performing qualitative and quantitative analysis of betalains. Semi-quantitative methods relied on measuring HPLC peak area relative to total peak areas [29,32]. This method requires suitable separation chromatography. Some researchers isolated the major betacyanin, betanin, and the betaxanthin, vulgaxanthin I, from beetroot extract and used them for semi-quantification of betacyanins as betanin equivalent and betaxanthins as betaxanthin I equivalent [34]. Others had to isolate major betacyanins and semi-synthesized betanin derivatives, and betaxanthins scale chromatographic purification of red beet root extract, allowed the production of more concentrated betalain formulations. Thirty betacyanins, including ten isomers, and seventeen betaxanthins with seven isomers were tentatively identified using HPLC-MS. Betanin/isobetanin represented 41% of total betacyanins. Others included decarboxylated betanin derivatives and neobetanin.

In another study of thirteen different varieties of beetroot, Sawicki [31] indicated that betanin, isobetanin, and vulgaxanthin I constituted the predominant compounds. Betanin and isobetanin represented 41–64% and 15–20%, respectively, while vulgaxanthin I ranged from 13.3 to 28.3% of total betalain content in the examined varieties. Compared to the previous studies [29,31], the LC-MS profile of our FD and SD particles indicated that the two encapsulation methods captured the main betalain components from beetroot extract.

### 3.2. Storage Stability

#### 3.2.1. Physicochemical Attributes—A*_w_* and Color

**Figure 4:**
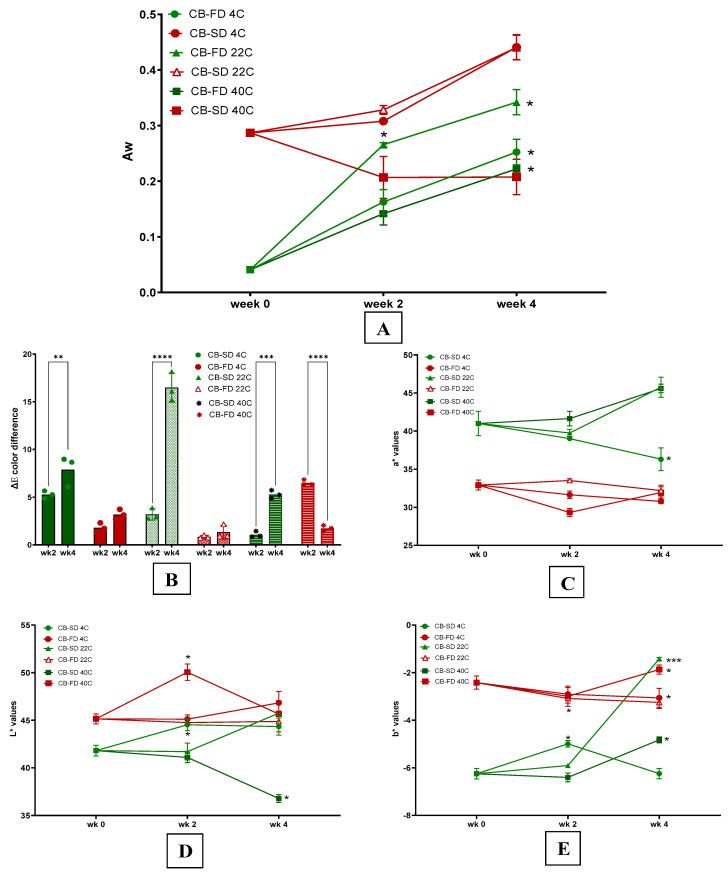
A*_w_* (**A**), ΔE total color difference (**B**), L* (**C**), a* (**D**), and b* (**E**) results of protein–betalain particles during 28-day storage at different temperatures. Legend: CB-FD 4C: freeze-dried chickpea protein–betalain particles stored at 4 °C; CB-FD 22C: freeze-dried chickpea protein–betalain particles stored at 22 °C; CB-FD 40 C: freeze-dried chickpea protein–betalain particles stored at 40 °C; CB-SD 4C: spray-dried chickpea protein–betalain particles stored at 4 °C; CB-SD 22C: spray-dried chickpea protein–betalain particles stored at 22 °C; CB-SD 40 C: spray-dried chickpea protein–betalain particles stored at 40 °C. Asterisks indicate significant statistical differences by ANOVA analysis and Dunnett’s test between samples analyzed after 2 or 4 weeks of storage and right after production (week 0). *: *p* ≤ 0.05; **: *p* ≤0.01; ***: *p* ≤ 0.001; ****: *p* ≤ 0.0001. For clarity, some asterisks are shown on the side of the experimental point.

Only freeze-dried particles had a significant A*_w_* increase during 4-week storage at different temperatures (Figure 4A). CB-FD had an initial lower A*_w_* than CB-SD, and therefore, the higher water gradient between the protein–betalain particles and the environment creates favorable conditions for water uptake. This tendency agrees with previous reports assessing food powders kept under similar storage conditions [35]. However, despite the observed variations during storage, all protein–betalain particles had A*_w_* levels within the microbiologically safe range [36].

The International Commission on Illumination (CIE) recommends the following interpretation of results: ΔE < 5: negligible color variation, 5 ≤ ΔE < 12: detectable color difference, and ΔE ≥ 12: remarkable color difference [37]. According to this scale, after 2 weeks of storage, the color differences were negligible, while after 4 weeks, higher values were detected but still under the threshold of ΔE < 12, except for CB-SD. Indeed, after 4 weeks, treatment CB-SD stored at 22 °C reached the highest ΔE, which coincides with higher a* and b* values (Figure 4C,E). Although most groups showed increased ΔE as storage progressed, CB-FD stored at 40 °C had higher ΔE after 2 weeks followed by a decrease after 4 weeks. This atypical behavior is justified by an abruptly higher L* after 2 weeks, followed by a significant decrease after 4 weeks (Figure 4D). Our ΔE results are lower than observed for betalain extracts produced from beetroots and stored at 4, 25, and 40 °C [37]. 

Moreover, results show a clear color differentiation between treatments. Independent of the temperature, during the 28-day storage, CB-SD particles maintained a more intense red color (higher a* values) and darker tones (lower L*) compared to CB-FD (Figure 4C,D). The a* values of CB-SD are higher than beetroot juice concentrate spray-dried with maltodextrin, gum Arabic, and whey protein concentrate [38], while our L* values are lower than encapsulated beetroot extracts produced by spray and freeze drying [20].

Red is the dominant color in beetroot extracts, and a* values are important color markers for protein–betalain particles. Interestingly, as storage progressed, the red color of CB-SD particles was maintained or intensified, as demonstrated by stable or increased a* values (Figure 4C). This finding is a positive indication of the stabilization of encapsulated natural beetroot pigments. Indeed, it has been shown that the degradation of betacyanins and betaxanthins can be minimized by exposing the extracts to mild temperatures around or below 60 °C [39]. Therefore, besides the effective betalains encapsulation by chickpea protein [38], the low outlet spray drying temperature (kept around 65 °C) and the short spray drying time [40] justify the observed preservation of protein–betalain particle redness.

#### 3.2.2. Thermal Stability of Betalains and Antioxidant Activity

**Figure 5:**
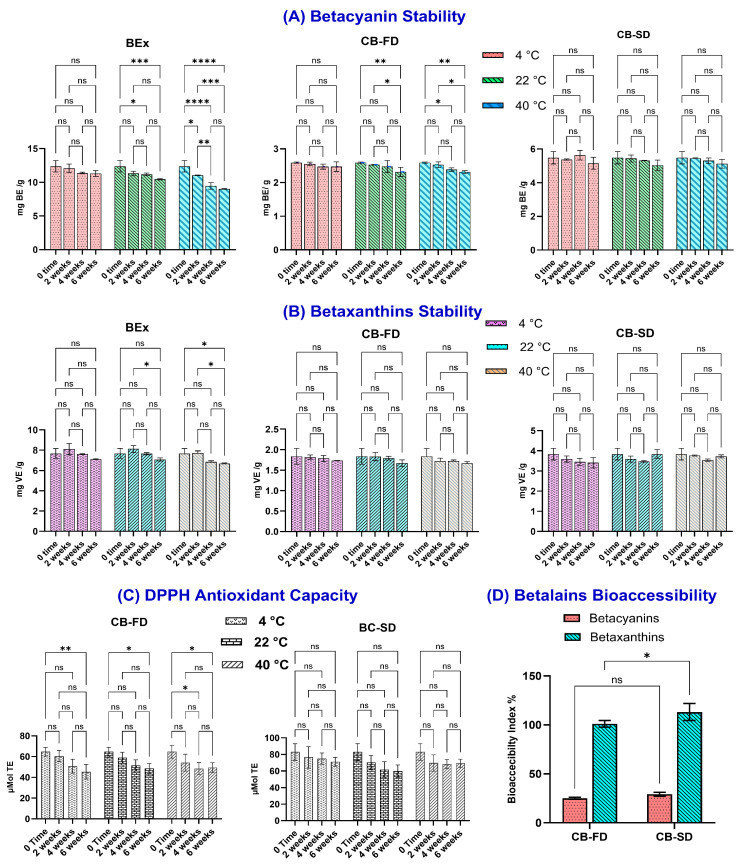
Retention of betacyanins (**A**), betaxanthins (**B**), and DPPH antioxidant capacity (**C**) of beetroot extract (BEx), freeze-dried chickpea protein–betalain particles (BC-FD) and spray-dried chickpea protein–betalain particles (CB-SD) during six weeks of storage at 5 °C, 22 °C, and 40 °C. (**D**) Bioaccessibility index of betacyanins and betaxanthins after intestinal phase digestion. According to ANOVA analysis and Dunnett’s test, asterisks indicate significant differences between samples analyzed after 2, 4, or 6 weeks of storage and right after production (week 0). *: *p* ≤ 0.05; **: *p* ≤0.01; ***: *p* ≤ 0.001; ****: *p* ≤ 0.0001.

The retention of betacyanins in CB-FD, CB-SD, and the non-encapsulated BEx remained consistent over a six-week period when stored at 4 °C (Figure 5A). In contrast, BEx exhibited significant degradation starting at the four-week mark (*p* > 0.5), with an increased decline at week six under 22 °C storage. The elevated temperature had a greater impact on BEx, maintaining only 73% betacyanin retention at 40 °C. On the other hand, the encapsulated forms, CB-FD and CB-SD, demonstrated superior stability compared to BEx, particularly CB-SD, which maintained its stability without significant changes throughout six weeks of storage at 4, 22, and 40 °C. While CB-FD showed some decrease in stability, with a slight drop in betacyanin content—retaining 89% after six weeks at both 22 °C and 40 °C—CB-SD remained the more stable option. Betaxanthin pigments in CB-FD and CB-SD exhibited remarkable stability, with no significant differences observed over six weeks (Figure 5B).

Despite a few limitations, the DPPH radical scavenging assay remains a popular method for assessing the antioxidant activities found in natural products. This assay is known for its simplicity, affordability, speed, reproducibility, and compatibility with thermally unstable compounds. Additionally, there is a strong correlation between the results and the content of bioactive compounds, with a regression factor exceeding 0.8 [41]. Some precautions were applied, like performing the assay in organic media and avoiding exposure to light. The DPPH radical scavenging activity results indicated a linear correlation coefficient with total betalain concentration (*r* = 0.86), shown in Figure 5C.

At higher temperatures, betalains undergo several degradation processes, including hydrolysis, isomerization, dehydrogenation, deglycosylation, and decarboxylation [42]. Specifically, increased heat prompts the decarboxylation of betanin, resulting in the formation of neobetanin, which alters the color due to creating a less stable aglycone [42]. Additionally, the thermal breakdown of betalains leads to the production of mono-, di-, and tricarboxylic betacyanins [32].

Pigment concentration plays a significant role in the thermal degradation of betalains. Increased pigmentation levels enhance betalains’ stability [43]. The low total betalain content in CB-FD (4.40 ± 0.29 mg/g), which is less than half the concentration found in CB-SD (9.34 ± 1.0 mg/g), slightly lowers the stability of CB-FD compared to CB-SD particles.

An increase in water activity (A*_w_*) can lead to the degradation of phytochemical compounds throughout storage. Water absorption and the resultant rise in water activity led to greater carrier plasticity and promoted phytochemical degradation, for instance, under identical bay losses in total phenolic content and anthocyanins [44]. Only CB-FD demonstrated a notable A*_w_* increase at 4 and 22 °C by the fourth week (Figure 5A). Initially, CB-FD had a lower A*_w_* than CB-SD, but it increased significantly over time, adversely impacting the stability of CB-FD.

Overall, our findings demonstrate the efficacy of chickpea encapsulation in prolonging the shelf-life of betalains, with spray drying being the most effective encapsulation method for beetroot extracts.

Many proteins have been proven to transport and enhance the stability of bioactives of small molecules. Soy protein encapsulated with beetroot extract by FD increased the thermal retention of betalain from 55.3% to 75.9%, as reported by Hu et al. [45]. In another citation, rice protein and pea protein significantly decreased the thermal degradation rate of betalain from 94% to 56%. They referred that to the hydrophobic forces and hydrogen bonding between protein and betalains [46].

#### 3.2.3. In Vitro Simulated Gastrointestinal Digestion

Bioaccessibility was assessed by measuring the contents of betacyanins and betaxanthins following in vitro simulated gastrointestinal digestion (SGD) of CB-FD and CB-SD particles. Figure 5D illustrates the % bioaccessibility of betacyanins and betaxanthins in the intestinal supernatant after SGD. We observed a loss of 74.9% of total betacyanins for CB-FD and 70.84% for CB-SD, with no significant differences, suggesting that betacyanins were less stable under digestion conditions. Previous reports noted even lower % bioaccessibility levels of betacyanins [47]. In another study, betacyanins was decreased by 22% during the simulated oral digestion, did not change in the gastric digestion stage, whereas a significant decrease was noted after the intestinal digestion step [48]. The reduction in betacyanins during SGD may be attributed to the hydrophobic nature of betanin, the primary component, coupled with digestive factors such as high temperatures, enzymes, and alterations in pH [31,47]. In contrast, betaxanthins displayed greater stability during SGD, achieving recovery rates ≥ 100%. This remarkable stability is in line with the previously mentioned storage stability and aligns with the research by Tesoriere [49]. Their findings show that betaxanthins were completely soluble in the aqueous (bioaccessible) fraction after the ultracentrifugation of post-intestinal digesta, while the release of betacyanins from the matrix was not fully achieved. Betacyanins are more sensitive to oxygen and temperature, and they display less stability in low pH environments compared to betaxanthins. In contrast, betaxanthins demonstrate greater stability in acidic conditions and during exposure to hydrolytic enzymes [49].

## 4. Conclusions

This study explored how encapsulating beetroot extract in chickpea protein isolate by freeze drying and spray drying affects various properties. CB-SD showcased a more vibrant and darker appearance than CB-FD, and different morphology was observed, with CB-SD being smaller, round-shaped particles and CB-FD having irregular fractured structures. CB-SD had higher A*_w_* and water solubility compared to CB-SD. Interestingly, both particles outperformed regular chickpea protein in terms of solubility.

CB-FD experienced a significant increase in A*_w_* at 4, 22, and 40 °C during storage, but results remained within microbiological safety limits. Betalains were more stable when encapsulated in spray-dried particles over a six-week period at 22 and 40 °C. The antioxidant capacity (DPPH) is strongly correlated with the betalain content. When subjected to simulated gastrointestinal digestion, there was a substantial loss in betacyanidin. However, betaxanthins demonstrated remarkable stability and retention following the intestinal phase of digestion. In summary, encapsulating betalain compounds from beetroot extract in chickpea protein isolate via spray drying is a clean, efficient, and cost-effective technology to produce food ingredients with preserved phytochemical content and extended shelf life. In future research, it will be important to explore additional components in beetroot, including phenolics, vitamins, and sugars, while assessing their bioaccessibility data. Furthermore, contrasting the results of this study with in vivo bioaccessibility data could yield significant insights.

## Figures and Tables

**Table 1:** Betalain retention and physicochemical properties of protein–betalain particles produced by freeze drying and spray drying.

Parameters	CB-FD	CB-SD
Moisture, %	1.66 ± 0.12 ^b^	4.65 ± 0.05 ^a^
Water activity (A*_w_*)	0.0436 ± 0.0043 ^b^	0.2868 ± 0.0024 ^a^
Hygroscopicity (% *w*/*w*)	14.90 ± 0.20 ^b^	18.85 ± 0.03 ^a^
Solubility (% *w*/*w*)	52.41 ± 0.94 ^b^	78.25 ± 0.58 ^a^
Instrumental color	L* = 44.33 ± 1.13 a* = 33.31 ± 0.82 b* = −2.63 ± 0.41	L* = 41.22 ± 1.09 a* = 42.83 ± 2.84 b* = −6.16 ± 0.23
Betalain Content		
Betacyanins (mg/g)	2.59 ± 0.12	5.48 ± 0.36
Betaxanthins (mg/g)	1.84 ± 0.19	3.84 ± 0.29
Total Betalains (mg/g)	4.40 ± 0.29	9.32 ± 1.51
DPPH Antioxidant capacity (µMol TE)	64.75 ± 4.2	82.95 ± 10.0

Results are shown as mean ± standard deviation. Sample identification: freeze-dried chickpea protein–betalain particles (CB-FD) and spray-dried chickpea protein–betalain particles (CB-SD). Different letters (^a^,^b^) on the same line indicate a significant difference using an unpaired *t*-test (*p* < 0.05).

**Table 2:** Betalain compounds identified in protein–betalain particles produced by freeze drying and spray drying from beetroot extract and chickpea protein isolate.

No.	Compound	Rt (min)	MS *m*/*z* [M + H]^+^	MS/MS *m*/*z*	FD-CB ^1^	SD-CB ^2^	References
Betacyanins (Betanins)							
1	Betanin	8.73	551.15	389	+	+	[29]
2	Betanidin	8.78	389.09	345	+	+	[29]
3	17-Decarboxybetanin	8.78	507.16	345	+	+	[29]
4	2,17-Bidecarboxy-neobetanin	8.78	461.15	299	+	+	[29]
5	17-Decaboxyneobetanin	8.80	505.14	343, 297	+	+	[29]
6	2′-*O*-Glucosyl-isobetanin	8.90	713.2	551, 389	+	+	[29]
7	Prebetanin	9.05	631.1	551, 389	+	+	[29]
3′	17-Decarboxyisobetanin	9.78	507.16	345	+	+	[29]
2′	Isobetanidin	9.95	389.09	345	+	+	[29]
1′	Isobetanin	9.96	551.15	389	+	+	[29]
10	Neobetanin	13.47	549.13	387	+	+	[29]
11	2-Decarboxy-neobetanin	14.19	505.14	343, 297	+	+	[29]
Betaxanthins (bx)							
12	Phenyl alanine-isobx	2.51	359.12	315	+	+	[29,30]
13	Asparagine-bx (Vulgaxanthin III)	2.59	326.1	280	+	+	[29,30]
14	Glutamine-bx (Vulgaxanthin I)	2.62	340.11	323, 277	+	+	[29,30]
15	Proline-bx (Indicaxanthin)	8.59	309.11	291	+	+	[29,30]
16	Valine-bx/-IsoBx	14.55	311.12	267	+	+	[29]
17	Leucine-bx (Vulgaxanthin IV)	18.72	325.14	281	+	+	[29]

^1^ Freeze-dried chickpea protein–betalain; ^2^ spray-dried chickpea protein–betalain particles.

## Data Availability

The original contributions presented in this study are included in the article/Supplementary Materials. Further inquiries can be directed to the corresponding author.

## Supplementary Materials

The following supporting information can be downloaded at: https://www.mdpi.com/article/10.3390/foods14020281/s1, Table S1. Betalain content in chickpea protein–beetroot extract (CP-BRE) mixture before and after spray drying at three protein concentrations: 6, 8, and 10%. Table S2. Betalain content in chickpea protein–beetroot extract (CP-BRE) after freeze drying at three protein concentration: 6, 8, and 10%.
