# Supplementary material for: Betalain–Chickpea Protein Particles Produced by Freeze Drying and Spray Drying: Physicochemical Aspects, Storage Stability, and In Vitro Digestion
Source: Foods. 2025 Jan 16;14(2):281. doi: 10.3390/foods14020281 (PMC11765016; doi:10.3390/foods14020281)
Supplement: Supplementary file 1 [file foods-14-00281-s001.zip › foods-3383667-supplementary.pdf]

Supplementary Information

Betalain–Chickpea Protein Particles Produced by Freeze Drying and Spray Drying: Physicochemical Aspects, Storage Stability, and In Vitro Digestion

Table S1. Betalain content in chickpea protein-beetroot extract (CP-BRE) mixture before and after spray drying at three protein concentrations; 6, 8, and 10%

	Betacyanins mg/g	Betaxanthins mg/g	Total Betalains mg/g
Betalain content in BRE, (2.5 °Bx)	5.49 ± 0.00	3.58 ± 0.00	9.08 ± 0.00
6% CP-BRE before SD	3.49 ± 0.02	2.56 ± 0.02	6.05 ± 0.00
6% CP-BRE after SD (CB-FD)	4.93 ± 0.01	3.95 ± 0.00	**8.87 ± 0.01**
8% CP-BRE before SD	3.09 ± 0.02	2.46 ± 0.01	5.56 ± 0.01
8% CP-BRE after SD	3.83 ± 0.01	3.95 ± 0.02	7.78 ± 0.01
10% CP-BRE before SD	3.03 ± 0.02	2.31 ± 0.01	5.33 ± 0.01
10% CP-BRE after SD	3.85 ± 0.02	2.88 ± 0.00	6.73 ± 0.02

Table S2. Betalain content in chickpea protein-beetroot extract CP-BRE after freeze drying at three protein concentration; 6, 8, and 10%

	Betacyanins mg/g	Betaxanthins mg/g	Total Betalains mg/g
Betalain content in beetroot extract, (2.5 °Bx)	5.49 ± 0.00	3.58 ± 0.00	9.08 ± 0.00
6% CP-BRE FD	2.56 ± 0.07	1.74 ± 0.04	**4.30 ± 0.11**
8% CP-BRE FD	2.42 ± 0.02	1.50 ± 0.00	3.93 ± 0.02
10% CP-BRE FD	2.02 ± 0.05	1.45 ± 0.00	3.48 ± 0.04
